# Supplementary material for: Lost in the crowd: Imagining walking in synchrony with a crowd increases affiliation and deindividuation
Source: PLoS One. 2021 Jul 23;16(7):e0254017. doi: 10.1371/journal.pone.0254017 (PMC8301649; doi:10.1371/journal.pone.0254017)
Supplement: S1 File — (DOCX) [file pone.0254017.s001.docx]

S1 File

Appendix 1

Principal Components Analysis for Task Difficulty Items

Principal components analysis (PCA) with oblique rotation (direct oblimin) was conducted on the 3 items assessing task difficulty. The Kaiser-Meyer-Olkin measure verified the sampling adequacy for the analysis, KMO = .57 which is above the acceptable limit of .5 (Field, 2009). Bartlett’s test of sphericity χ^2^ (3) = 220.80, *p* < .001, indicated that correlations between items were sufficiently large for PCA. One component was extracted. This had an eigenvalue over Kaiser’s criterion of 1 and explained 58.56% of the variance. Table 2 shows factor loadings after rotation.

Table 2. Summary of factor loadings for task difficulty items.

|  | Factor Loadings |
| --- | --- |
| Item | Task Difficulty |
| Successfulness | .86 |
| Vividness | .78 |
| Difficulty | .64 |
| Eigenvalues | 1.76 |
| % of variance | 58.56 |

Appendix 2

Principal Components Analysis for Affiliation Items

Principal components analysis (PCA) with oblique rotation (direct oblimin) was conducted on the 8 items assessing affiliation. The Kaiser-Meyer-Olkin measure verified the sampling adequacy for the analysis, KMO = .92 (‘superb’ according to Field, 2009). Bartlett’s test of sphericity χ^2^ (28) = 1521.33, *p* < .001, indicated that correlations between items were sufficiently large for PCA. Only one component was extracted. This had an eigenvalue over Kaiser’s criterion of 1 and explained 54.19% of the variance. Table 3 shows factor loadings after rotation.

Table 3. Summary of factor loadings for affiliation items.

|  | Factor Loadings |
| --- | --- |
| Item | Affiliation |
| Closeness | .76 |
| Connectedness | .80 |
| Liking | .79 |
| Trust | .73 |
| Rapport | .75 |
| Similarity | .59 |
| Would wish to see again | .75 |
| Would wish to get to know them | .70 |
| Eigenvalues | 4.34 |
| % of variance | 54.19 |

Appendix 3

Principal Components Analysis for Deindividuation Items

Principal components analysis (PCA) with oblique rotation (direct oblimin) was conducted on the 3 items assessing task difficulty. The Kaiser-Meyer-Olkin measure verified the sampling adequacy for the analysis, KMO = .51 which is above the acceptable limit of .5 (Field, 2009). Bartlett’s test of sphericity χ^2^ (3) = 168.58, *p* < .001, indicated that correlations between items were sufficiently large for PCA. One component was extracted. This had an eigenvalue over Kaiser’s criterion of 1 and explained 53.56% of the variance. Table 4 shows factor loadings after rotation.

Table 4. Summary of factor loadings for deindividuation items.

|  | Factor Loadings |
| --- | --- |
| Item | Deindividuation |
| Individual | .86 |
| Group member | .79 |
| Individual-group member | .49 |
| Eigenvalues | 1.61 |
| % of variance | 53.56 |
