# Supplementary material for: Lost in the crowd: Imagining walking in synchrony with a crowd increases affiliation and deindividuation
Source: PLoS One. 2021 Jul 23;16(7):e0254017. doi: 10.1371/journal.pone.0254017 (PMC8301649; doi:10.1371/journal.pone.0254017)
Supplement: Attachment — Submitted filename: Response to reviewers.docx [file pone.0254017.s002.docx]

Dear Dr Wallot,

Please find attached a revised manuscript following our careful attention to the very helpful comments provided by the reviewers. We believe that the revisions have significantly improved the manuscript and hope that it is now acceptable for publication in PLOS ONE. We would also like to take this opportunity to thanSuoplmentarymeatielas k both reviewers for their time and comments in helping us improve this work.

We look forward to hearing from you.

With kind regards.

Yours sincerely,

The authors.

**Reviewer #1:** Dear editor,
I have now reviewed the paper entitled “Lost in the crowd: Imagining walking in synchrony with a crowd increases affiliation and deindividuation”. In the paper, authors asked participants to imagine walking in a crowd in either in coordination or in an uncoordinated manner. They compared the impact of imagined synchronous and non-synchronous movement in a crowd on affiliation and deindividuation. Overall, the paper is well-written and easy to follow. The theoretical background is presented well and the research is grounded on previous work clearly. The methodology followed in the study is capable of answering the research questions stated in the paper. Results and discussion sections are organized comprehensively.
I have few concerns regarding some aspects of the paper:

Comments:

**1. The main starting point of the paper is that in real-life group synchrony might be influenced by cultural primers such as beliefs, ideologies and dressing. In order to control the impact of cultural confounds on group synchrony, authors conducted an online experiment and asked participants to imagine walking in a crowd. In the paper, authors claim that such experimental design would not be confounded by cultural aspects. In the experiment, participants were asked to imagine themselves walking in a crowd. Researchers did not specify a specific crowd. Thus, participants were free to imagine themselves in any kind of group. In one way, this design might considered as a rigorous approach to avoid confounds about group culture. In other way, it can be seen as a limitation: What if the participants considered the groups they are affiliated most when imagining walking in coordination or not? For example, a participant from an army background might have imagined walking with soldiers, a doctor might have imagined walking with other doctors, and so on. I wonder asking participants to imagine walking with “complete strangers” in a crowd would have been a better approach to answer the research questions stated in the paper?**

*We thank the reviewer for this comment, the reviewer is indeed right that we didn’t specify any crowd characteristics, and people might have imagined crowds of friends/ co-workers, platoon members etc. That said this would be the case across both conditions equally. The fact we show an effect post experimental condition compared to control leaves us confident it was indeed the imagined synchrony that we accounted for this effect. Still we take your point that this initial framing may have been misleading and unhelpful and have amended this accordingly in the manuscript. It has been removed form the intro and discussed more thoroughly in the discussion in line with your concern. Thank you for pointing this out to us.*

**2. In terms of self-reported instruments, authors do not present any statistical findings regarding the validity and/or reliability of those instruments. I am not much familiar with this field. Thus, I wonder if it is OK to ignore the reliability/validity of scales in this line of research?**

*We thank the reviewer for noting this omission and we have now have included scale analysis on our measures as requested. , This can be found in the supplementary materials.*

**3. In the Discussion authors claim that “…imagined synchrony interventions can be used to improve the sense of social connectedness for individuals experiencing loneliness and social isolation, and those who are unable to physically coordinate.” I think authors should present some strong arguments/evidence on this practical implication. As far as I know, social relatedness is about seeing one’s self as part of a specific social group (e.g. family, tribe, and team). How can one deal with social loneliness by imagining being in synch with a group that has no cultural relevance to him/her? How can a social system exist without common cultural features? I understand authors point in exploring how synchrony manifests itself when cultural features are somehow wiped out. However, I am not sure the current findings are applicable to the real-life settings where cultural norms and features have inevitable components of groups.**

*We believe the section highlighted by the reviewer is indeed too strong a claim and simply over steps the dat we have available. We have therefore removed this claim from the resubmission.*

**Reviewer #2:** The authors investigate whether imaging walking in synchrony with a crowd promotes in the participants feelings of deindividuation and affiliation with the crowd compared to imaging walking asynchronously, as well as whether individuation mediates the effect of imagined synchrony on affiliation.

In contrast with a large part of the studies available in the literature, the study focuses on the embodied elements of deindividuation rather than on the cognitive elements, and it tackles the interesting issue of whether imagined synchrony produces the same effect as physical synchronous actions with respect to feelings of deindividuation and affiliation.
It is the first study to show the positive effect of imagining walking in synchrony on affiliation and, as the authors suggests, it opens paths to future (online) research developments on important topics, including social connectedness and the reduction of prejudicial attitudes. Because of this it can be considered of high relevance and originality. Because of this, I recommend it for publication.

However, some minor revisions might contribute to the quality of the manuscript.

**Comments:

1. In the introductory section, the authors go from discussing contemporary research on deindividuation and coordination to the theorizing of McNeill (1995) and Durkheim (1912) to then go back to the contemporary research. I would suggest restructuring the section following a chronological order to facilitate the reading.**

*We would like to thank the reviewer for highlighting this issue. We have rewritten the introduction in order to present our rationale more clearly. Unfortunately we have been unable to present the information chronologically throughout as we found in trying to do this, we were jumping from one topic to another and back again which further disjointed the flow. That said we believe that our rewritten introduction solves the fundamental confusion that the reviewer rightly highlights. In the new introduction, rather than merely highlighting McNeill’s view that the interaction between endorphins and experience may give rise to the social and cognitive effects of interpersonal synchrony, we have explained why it is that this presents a problem for synchrony research, and have highlighted that this will be addressed by our research. We hope that our introduction now clarifies this point, and clarifies the rationale for the research.*

**2. Still in the introductory section, the discussion of what it means for crowds to have “personalities” could also be better organized, as the concerning content of the first page seems to be partially repeated on page 4 and 5.**

*In the rewritten version of the introduction we have now removed the repetition of this point. We also feel that the term “personality”, which usually refers to individual rather than group differences and exists as a field of research in its own right, distracts somewhat from the arguments that we were making and have therefore opted instead to use the term “characteristics”.* *We again thank the reviewer for pointing this out.*

**3. In the discussion, the authors claim that “it is important to point out that even though deindividuation may affect affiliation, and affiliation increases pro-sociality, deindividuation does not necessarily always lead to prosocial outcomes.” As the discussion goes, it seems to be suggested that deindividuation promotes feelings of affiliation toward the in-group while potentially leading to even anti-social behaviors toward the out-group. Thus, the important point would be that deindividuation promotes affiliation toward the in-group but not (necessarily) toward out-groups. This point could be better clarified by the authors.**

*This is an excellent point. We have now added the following to our discussion of this issue in order to ensure that the reader better understands this: “That is, deindividuation promotes feelings of affiliation with the ingroup while often also resulting in feelings of antipathy and antisocial behaviour directed at outgroup members.”*

**4. The cited paper authored by Herrera (2018) is missing in the references.**

*Thanks for spotting this, We have now added the reference to the reference section and double checked all citations are present.*

*“Herrera, E. (2018). Masculinity, violence, and deindividuation in Argentine soccer chants: the sonic potentials of participatory sounding-in-synchrony. Ethnomusicology, 62(3), 470-499.”*
